# Reliable and accurate prediction of basic pK_a_ values in nitrogen compounds: the pK_a_ shift in supramolecular systems as a case study

**DOI:** 10.1186/s13321-023-00763-3

**Published:** 2023-09-28

**Authors:** Jackson J. Alcázar, Alessandra C. Misad Saide, Paola R. Campodónico

**Affiliations:** 1 Centro de Química Médica, Universidad del Desarrollo, Av.Plaza 680, 7780272 Santiago, RM Chile; 2 Santiago, RM Chile

**Keywords:** Acidity, QSAR, Quantum descriptors, DFT, Cucurbiturils

## Abstract

This article presents a quantitative structure–activity relationship (QSAR) approach for predicting the acid dissociation constant (pK$_a$) of nitrogenous compounds, including those within supramolecular complexes based on cucurbiturils. The model combines low-cost quantum mechanical calculations with QSAR methodology and linear regressions to achieve accurate predictions for a broad range of nitrogen-containing compounds. The model was developed using a diverse dataset of 130 nitrogenous compounds and exhibits excellent predictive performance, with a high coefficient of determination (R$^2$) of 0.9905, low standard error (*s*) of 0.3066, and high Fisher statistic (*F*) of 2142. The model outperforms existing methods, such as Chemaxon software and previous studies, in terms of accuracy and its ability to handle heterogeneous datasets. External validation on pharmaceutical ingredients, dyes, and supramolecular complexes based on cucurbiturils confirms the reliability of the model. To enhance usability, a script-like tool has been developed, providing a streamlined process for users to access the model. This study represents a significant advancement in pK$_a$ prediction, offering valuable insights for drug design and supramolecular system optimization.

**Graphical Abstract:**

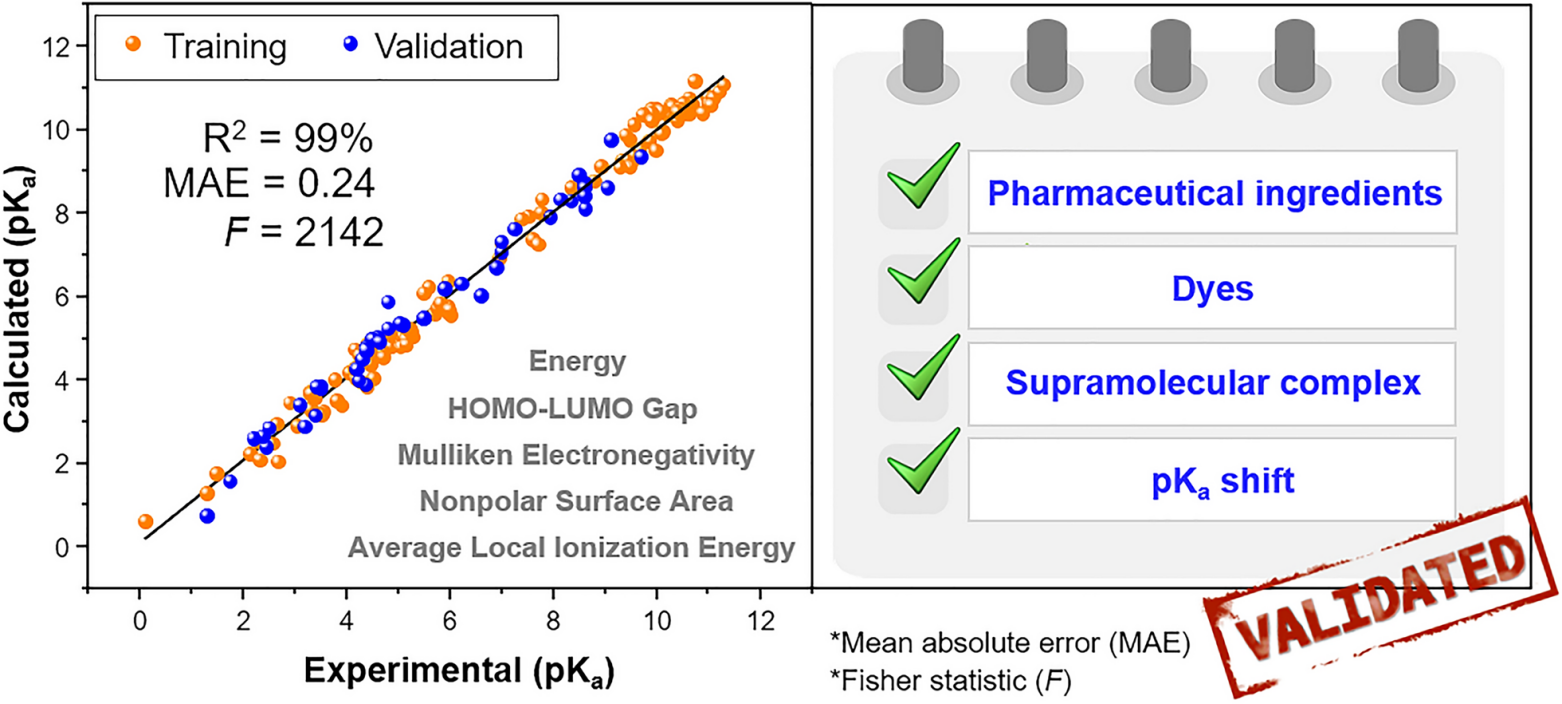

**Supplementary Information:**

The online version contains supplementary material available at 10.1186/s13321-023-00763-3.

## Introduction

The concepts of acidity and basicity are fundamental to the understanding of chemistry and have been defined by various theories throughout history [2, 18, 19, 54, 55]. One such theory was introduced by Svante Arrhenius in 1887 [2], who suggested that certain compounds can dissociate into ions in solution and identified acids as those that yield a proton (H$^+$) and bases as those that yield a hydroxide ion (OH$^-$). Another influential theory, the General Acid–Base Theory of Brönsted and Lowry [18, 19, 55], emerged in 1923, defining acidity and basicity in terms of the tendency to donate or accept a H$^+$.

Understanding the strength of a base is crucial in comprehending its behavior and acidity plays a pivotal role in this regard. The strength of a base is commonly expressed by considering the strength of its conjugated acid, with a weaker conjugated acid indicating a stronger base. The acidity constant (K$_a$), which represents the equilibrium constant for the reaction between the acid and water, is used to quantitatively assess the strength of a base. For practical purposes, the pK$_a$ value, defined as the negative logarithm of the K$_a$, is commonly employed [40]. The pK$_a$ value is an essential tool that serves as an indicator of the relative acidity or basicity of a compound and enables predictions of its protonation state or protomeric forms under different pH conditions [40]. Therefore, accurate determination of pK$_a$ holds immense significance across diverse fields, including medicinal chemistry [22, 29, 41, 60], biochemistry [11, 23, 62, 68], environmental science [12, 15, 48, 50], chemistry of dyes [44, 65, 93] and supramolecular chemistry [10, 56, 88].

Recent research has focused on the phenomenon of supramolecular pK$_a$ shift, which involves a significant shift in the pK$_a$ value of nitrogenous compounds by forming supramolecular complexes with macrocyclic molecules such as cucurbiturils [7, 8, 14, 59, 64, 95]. This phenomenon is crucial for designing and optimizing supramolecular systems, with far-reaching implications in materials science [92, 98], catalysis [3, 28, 77] and the development of drugs and their delivery methods [26, 31, 37, 49, 75].

In the pharmaceutical industry, accurate pK$_a$ values play a critical role in the design process of new drugs, as the acid/base character of a substance defines its biopharmaceutical properties, which have a direct impact on the pharmaceutical formulation of the drug [60, 61]. Nitrogen-containing heterocycles are of particular interest in the pharmaceutical industry due to their diverse biological activities [52, 53], with over 75$\%$ of FDA-approved drugs containing such structures [47]. The pK$_a$ values of these heterocycles provide information on the absorption, distribution, metabolism, excretion, and toxicity (ADMET) of the drug, which are crucial for the design process. Poor ADMET properties have been revealed as the cause for high attrition in the development phase [84].

Experimental methodologies for pK$_a$ determination have been extensively reviewed [74], but there are still samples that are difficult or impossible to measure accurately. To overcome this challenge, computational approaches have emerged as a promising alternative, as they can simulate virtually any set of working conditions without requiring physical samples [81, 83, 94]. Consequently, significant efforts have been directed toward developing accurate and reliable computational methods for predicting pK$_a$ values.

Among the most common computational approaches are First Principles and quantitative structure–activity relationship (QSAR) methods [81]. However, the accuracy of First Principles calculations in predicting pK$_a$ values relies heavily on the precise determination of the Gibbs free energy difference in solution, which poses a significant challenge [27, 73, 81]. This is mainly due to the difficulty in calculating the Gibbs free energy of the proton and solvation energies, which can lead to deviations in pK$_a$ values of up to 3 units [13, 73]. One way to address these systematic errors is by using the relative pK$_a$ approach [73, 81], which has demonstrated high accuracy and effectiveness in various solvents [83]. Nevertheless, this approach is limited by the availability of accurate pK$_a$ values for reference systems with structural similarity to the sample.

QSAR is a time-efficient and computationally less costly approach that predicts physical properties by constructing a multiple linear regression equation for a specific physical property as a function (*P*) of the selected molecular descriptors ($X_i$). The equation, represented as Eq. 1, assigns numerical coefficients ($a_i$) to each molecular descriptor, which serve as weighting factors to determine the respective contributions of the predictor variables [25, 81].

1 $$\begin{aligned} P = a_0 + a_{1}X_{1} + a_{2}X_{2} \end{aligned}$$

Although QSAR models are less costly than First Principles, traditional QSAR methods have been hindered by lengthy calculation times, especially when quantum-mechanical electronic descriptors are involved, particularly in large molecules. As a result, the prediction of pK$_a$ has been impractical. However, the B97-3c method, based on density functional theory (DFT), has recently emerged as a reliable and low-cost solution [16]. This method effectively reduces computational time, thereby presenting a viable option for predicting pK$_a$ values in large molecules and supramolecular complexes.

QSAR models have extensively employed a wide range of descriptors to predict crucial properties, including pK$_a$ values [36, 42, 45, 46, 70, 76, 79, 81, 90]. Some of these descriptors include charge [39, 45, 79], electronic energy differences ($\Delta$E) [5, 6, 42, 79, 97], and the highest occupied molecular orbital energy ($\epsilon _{HOMO}$) [80, 86]. Despite the flexibility in choosing chemical descriptors and their combinations, reported QSAR models are limited to specific datasets or structures, resulting in varying levels of accuracy and model performance across different subsets of data. Such is the case of QSAR models for predicting pK$_a$ values [96], which show diminished accuracy when predicting pK$_a$ of basic compounds such as nitrogenous compounds [78]. These discrepancies may arise due to the lack of appropriate relationship or representation between the selected descriptors and the structural diversity present in the complete dataset. Consequently, developing reliable QSAR models for predicting pK$_a$ values in heterogeneous datasets has proven to be a persistent challenge. To improve the generalizability of QSAR models and achieve higher precision in predicting pK$_a$ values, a more careful selection of chemical descriptors that effectively capture the structural variability within the entire dataset is necessary. This may involve the use of more specific descriptors or advanced variable selection methods.

The present incapacity to efficiently and accurately predict basic pK$_a$ values from heterogeneous data highlights the need for enhanced QSAR methodologies capable of achieving high accuracy regardless of the size and structure of the compound, as well as its inclusion in supramolecular host-guest systems. In light of this challenge, we propose and validate a comprehensive QSAR approach that utilizes the B97-3C low-cost density functional theory to predict the basic pK$_a$ values of nitrogen-containing compounds in aqueous solution at 25 $^{\circ }$C, both independently and within cucurbituril-based supramolecular complexes. This proposed approach represents a significant advancement toward predicting pK$_a$ shifts in supramolecular systems.

## Results and discussion

### The QSAR model

By employing a comprehensive approach that integrates DFT [30], Conceptual Density Functional Theory (CDFT) [30], Molecular Electron Density Theory (MEDT) [24], and quantitative analysis of molecular surface, prediction models for estimating pK$_a$ values were evaluated through statistical analysis. The best performing model was selected based on the results of this evaluation (see “Methods” section)

The selected prediction model is represented in Eq. 2.^1^ The model was trained using a diverse set of 130 nitrogenous compounds, which encompassed aromatic and non-aromatic cyclic amines as well as aliphatic amines (primary, secondary, and tertiary). A coefficient of determination (R$^2$) of 0.9905 indicates an excellent fit of the data to the proposed model. The robustness of the model is further supported by a high Fisher statistic (*F*) of 2141.9289 and a relatively low standard error (*s*) of 0.3066. The root mean squared error (RMSE), 0.2982, and mean absolute error (MAE), 0.2440, provide additional evidence of the predictive accuracy of the model.

2 $$\begin{aligned} & pK_a = 0.1074 \Delta E - 0.1422 \Delta HL_{Gap} - 0.9132\chi _M + 0.0151 \% NPSA - 1.4887 \Delta ALIE_N + 3.0608 BaseT - 30.7139 \nonumber \\& \quad n = 130; R^2 = 0.9905; s = 0.3066; F = 2141.9289; RMSE = 0.2982; MAE = 0.2440 \end{aligned}$$

Our model (Eq. 2) includes a variety of descriptors calculated in a vacuum environment and demonstrates remarkable performance when applied to a diverse set of nitrogenous compounds. The descriptors included in the model are the following:

- Energy of deprotonation ($\Delta$E) in kcal/mol: this descriptor measures the energy required to remove a proton from an acid. A higher $\Delta$E value signifies that a greater amount of energy is needed to carry out the deprotonation, which results in a higher pK$_a$ value.
- HOMO–LUMO gap of deprotonation ($\Delta$HL$_{Gap}$) in eV: this descriptor represents the change of energy gap between the highest occupied and lowest unoccupied orbitals of the acid–base equilibrium. A higher $\Delta$HL$_{Gap}$ suggests a less reactive base, hence a lower pK$_a$ value.
- Mulliken electronegativity ($\chi _M$) in eV: this descriptor quantifies the ability of the base to donate a pair of electrons and accept a proton. A lower $\chi _M$ indicates a higher basicity, which contributes to a higher pK$_a$ value.
- Nonpolar surface area percentage ($\%$NPSA) of the base: this descriptor measures hydrophobicity and its influence on the solubility of the base in water. Bases with a higher $\%$NPSA tend to have lower solubility, which leads to increased stability of the conjugated acid in a polar environment compared to the base. This reduced solubility directly affects the ability of the base to donate and accept protons, thus altering the acid–base equilibrium and resulting in an increase in the pK$_a$ value. Notably, the $\%$NPSA descriptor demonstrates one of the most significant individual correlations with the experimental pK$_a$ values, as evidenced by its correlation coefficient (r) of 0.7514 (refer to Additional file 1: Table S1).
- Change in average local ionization energy ($\Delta$ALIE$_N$): this descriptor quantifies the energy difference (in eV) required to remove an electron from the nitrogen atom in the acid–base reaction center. A smaller $\Delta$ALIE$_N$ indicates greater stabilization of the positive charge in the acid, leading to an increased pK$_a$ value
- Base type (BaseT): this categorical descriptor takes a value of 0 for aromatic amines and 1 for aliphatic or non-aromatic amines.

By considering such a comprehensive range of independent parameters (with a correlation between parameters $\le$ |0.7744|, see Additional file 1: Table S2), our model provides valuable insights into the electronic structure, stability, solubility, hydrophobicity, and local electronic effects during proton transfer in the bases under investigation. This holistic approach contributes to the accurate prediction of pK$_a$ values and enhances our understanding of the underlying factors governing acid–base behavior in nitrogenous compounds.

In comparison with the trading software Chemaxon, our model shows a higher R$^2$ (0.9905 versus 0.9583), lower s (0.3066 versus 0.6346), and lower RMSE and MAE (see Additional file 1: Table S3). These results demonstrate the superiority of the presented approach over the widely used Chemaxon software.

**Fig. 1:**
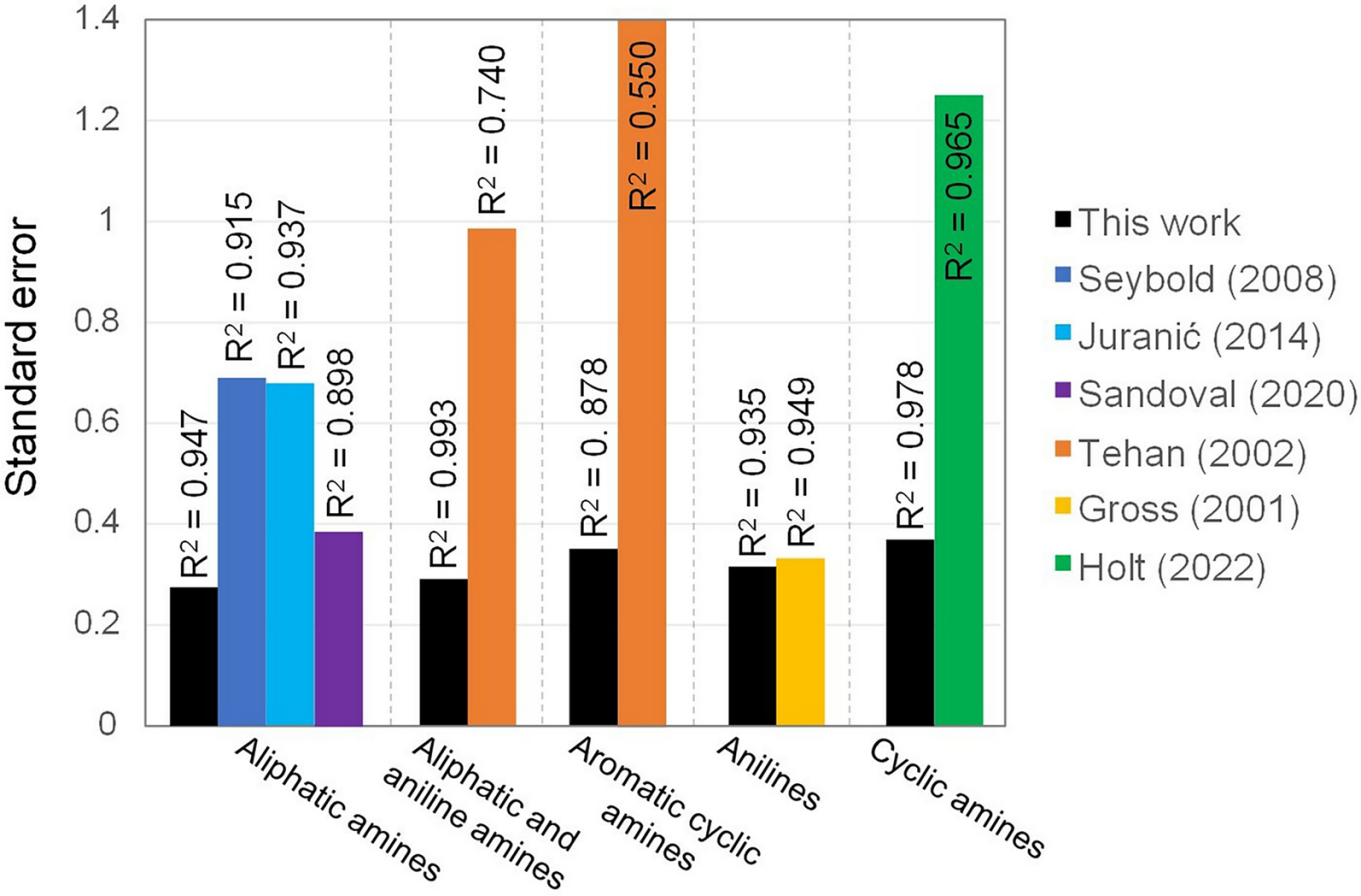
Comparative performance of our model against previous QSAR methods [36, 42, 45, 76, 79, 90] based on R$^2$ values and standard errors (*s*)

Regarding the comparison of our model with the QSAR studies previously reported [36, 42, 45, 76, 79, 90], it is necessary to note that our approach surpasses all the explored QSAR methods in terms of accuracy and versatility. Namely, Tehan’s [90] semi-empirical method using AM1, Seybold’s [79] method based on RM1, Juranić’s [45] approach with PM6, Gross’s [36] method using Hartree-Fock (HF)/6-311 G(d,p), Sandoval-Lira’s [76] DFT method based on $\omega$B97X-D/cc-pVDZ, and Holt’s [42] approach with B3LYP/6-31+G(d,p). Except for Gross’s [36] method, which performs similarly to ours for anilines, all methods reported lower R$^2$ values and higher standard errors (see Fig. 1).

While the cited works focused on a particular class of amines (non-heterogeneous data) and selected up to three parameters, we have expanded the predictive capabilities to a broader set of nitrogenous compounds by incorporating a comprehensive range of descriptors. This approach provides a more accurate estimation of the pK$_a$ value and greatly expands the range of applicability of our model.

Yu et al. [96] presented a comparative performance of ACD and SPARC software. While the statistical results of both software are similar to those of the present work, it is important to note that these software are commercial and employ proprietary methodologies, which may limit their accessibility and customization. A key advantage of our model is its generalizability to diverse types of amines and its ability to handle heterogeneous data, which is a common challenge in real-world applications.

### Model validation

#### External validation

The performance of our prediction model (Eq. 2), in estimating pK$_a$ values, was evaluated a total of 40 compounds, which were part of an external validation dataset comprising pharmaceutical ingredients and dyes. It is important to note that the 40 compounds in the external validation dataset are not part of the training dataset of 130 compounds. Additionally, our method was tested on 6 cucurbituril-based supramolecular complexes, which were not included in either of the two previous datasets. These results are summarized in Tables 1 and 2, respectively.

**Table 1:** Experimental and predicted basic pK$_a$ values for nitrogen compounds in pharmaceutical ingredients and dyes at 25 $^{\circ }$C in aqueous solution

ID	Nitrogenous compounds	Experimental pK$_a$	Predicted pK$_a$^a^	|Error|	Chemaxon pK$_a$ ^b^	|Error|	References
131	Norephedrine	9.12	9.72	0.60	8.96	0.16	[82]
132	Procaine	9.05	8.59	0.46	8.96	0.09	[82]
133	Terbutaline	8.62	8.14	0.48	9.76	1.14	[91]
134	Quinine	8.60	8.69	0.09	8.55	0.05	[91]
135	Tetracaine	8.50	8.87	0.37	8.42	0.08	[82]
136	Astemizole	8.35	8.25	0.10	9.23	0.88	[91]
137	Nicotine	8.14	8.32	0.18	8.58	0.44	[82]
138	Clozapine	7.94	7.85	0.09	8.16	0.22	[91]
139	Ampicillin	7.25	7.60	0.35	7.23	0.02	[78]
140	Ketoconazole	6.22	6.25	0.03	6.26	0.04	[91]
141	Clotrimazole	5.89	6.17	0.28	6.42	0.53	[43]
142	Benzimidazole	5.50	5.47	0.03	5.79	0.29	[67]
143	Coumarin 7	5.10	5.26	0.16	4.21	0.89	[9]
144	Abacavir	5.04	5.35	0.31	6.87	1.83	[82]
145	Fuberidazole	4.80	5.21	0.41	3.93	0.87	[51]
146	Thiabendazole	4.60	5.00	0.40	4.08	0.52	[51]
147	Carbendazim	4.50	4.98	0.48	4.28	0.22	[51]
148	Omeprazole	4.40	4.71	0.31	4.77	0.37	[91]
149	Cerivastatin	4.38	4.79	0.41	5.58	1.20	[91]
150	Ethionamide	4.37	3.89	0.48	5.00	0.63	[91]
151	Olmesartan medoxomil	4.30	4.48	0.18	3.65	0.65	[4]
152	Lamivudine	4.24	4.01	0.23	2.00	2.24	[82]
153	Picoprazole	3.50	3.78	0.28	2.89	0.61	[63]
154	2-Amino-pentamethylbodipy	3.50	3.77	0.27	0.41	3.09	[38]
155	Diazepam	3.42	3.84	0.42	2.92	0.50	[89]
156	Prodan	3.40	3.14	0.26	4.94	1.54	[21]
157	Timoprazole	3.10	3.41	0.31	2.38	0.72	[63]
158	Benzocaine	2.45	2.39	0.06	2.78	0.33	[91]
159	Metronidazole	2.38	2.65	0.27	3.03	0.65	[82]
160	Acyclovir	2.20	2.62	0.42	2.94	0.74	[82]
161	Sulfacetamide	1.75	1.58	0.17	2.14	0.39	[67]
162	7-Dimethylazacoumarin^c^	1.30	0.73	0.57	3.67	2.37	[1]
163	CHEMBL1689126^c^	7.00	7.07	0.07	6.54	0.46	[63]
164	CHEMBL1689112^c^	6.90	6.68	0.22	5.94	0.96	[63]
165	CHEMBL47529^c^	5.50	5.48	0.02	4.96	0.54	[63]
166	CHEMBL1349378^c^	4.64	4.90	0.26	4.49	0.15	[63]
167	CHEMBL432733^c^	4.37	4.72	0.35	7.39	3.02	[63]
168	CHEMBL271703^c^	4.20	4.26	0.06	3.46	0.74	[63]
169	CHEMBL191553^c^	3.20	2.89	0.31	2.80	0.40	[63]
170	CHEMBL1405150^c^	2.50	2.86	0.36	3.64	1.14	[63]
			MAE_ext:	0.28	MAE_ext:	0.60	

^a^Calculated by Eq. 2. RMSE_ext = 0.32

^b^Calculated by Chemaxon software. RMSE_ext = 1.09

^c^Does not have a common or short name. The IUPAC name is found in Additional file 1

**Table 2:** Predicted pK$_a$ values of nitrogen compounds in CB7-complexed states at 25$^{\circ }$C in aqueous solution (Predicted pK$_a$ $^{CB7}$) and their respective pK$_a$ shift

ID	Nitrogenous compounds	Exp.	Predicted^a^	|Error|	Exp.	Predicted^b^	|Error|	References
		pK$_a$ $^{CB7}$	pK$_a$ $^{CB7}$		pK$_a$	pK$_a$		
					Shift	Shift		
C1	Coumarin 7	9.70	9.36	0.34	4.60	4.06	0.54	[9]
C2	Thiabendazole	8.60	8.62	0.02	3.96	3.60	0.36	[51]
C3	Fuberidazole	8.60	8.41	0.19	3.80	3.17	0.63	[51]
C4	Carbendazim	7.00	7.31	0.31	2.50	2.35	0.15	[51]
C5	Prodan	6.60	6.02	0.58	3.20	2.84	0.36	[21]
C6	2-Amino-pentamethylbodipy	4.80	5.87	1.07	1.30	2.03	0.73	[38]
		MAE_ext:	0.42		MAE_ext:	0.46		

^a^Calculated by Eq. 2. RMSE_ext = 0.54

^b^Calculated pK$_a$ Shift using the Eq. 2 for both the free and complexed substrate. RMSE_ext = 0.50

According to data in Table 1, our model achieved a low external RMSE (RMSE_ext) of 0.32 and an external MAE (MAE_ext) of 0.28 when predicting pK$_a$ values for the external validation dataset of 40 pharmaceutical ingredients and dyes. Comparing these values with the corresponding metrics obtained from Chemaxon (RMSE_ext of 1.09 and a MAE_ext of 0.79), it is evident that our model outperforms Chemaxon in terms of accuracy and precision in predicting pK$_a$ values, especially when estimating pK$_a$ for supramolecular complexes, for which Chemaxon is currently incompatible.

For the dataset of 6 cucurbituril-based supramolecular complexes (Table 2), our model exhibited a slightly higher RMSE_ext of 0.54, indicating a moderate average deviation of the predicted pK$_a$ values for this dataset. Similarly, the MAE_ext value of 0.42 suggests a moderate average error of the estimated pK$_a$ values for the supramolecular complexes.

#### Internal validation

Furthermore, an internal validation assessment corroborates the excellent performance of our model. For internal validation within the training set of 130 compounds, we employed the “leave-one-out” cross-validation model. The small difference between the coefficient of determination (R$^2$) and the leave-one-out cross-validation correlation coefficient (Q$^2$loo), indicated by the stability value of 0.0013, attests to the robustness of our model and suggests that it is not overfitted.

These results validate the effectiveness and reliability of our prediction model in estimating basic pK$_a$ values of nitrogen-containing compounds, both as isolated species and as guests in cucurbiturils complexes. Further optimization and refinement of the model may enhance its performance in predicting pK$_a$ values for diverse chemical systems, extending beyond nitrogenous compounds.

### Script-like tool description

To enhance user experience with our model, we have developed a script-like tool that automates the determination of descriptors and pK$_a$ values for nitrogenous compounds. Users can easily access the estimation process through our tool by inputting the structures of the base and the conjugate acid. The tool is available at the following link: https://github.com/Jacksonalcazar/Basic-pKa-Estimation-Nitrogen-Compounds. This streamlined process provided by our tool offers users a more convenient and efficient way to utilize our model.

## Conclusion

The comprehensive QSAR approach presented in this article offers a powerful tool for rapidly and accurately predicting pK$_a$ values of nitrogenous compounds, including those within supramolecular complexes based on cucurbiturils. Our model, which combines quantum mechanical calculations and QSAR methodology, exhibits excellent predictive performance and provides valuable insights into various molecular properties relevant to proton transfer. The superiority of our approach over existing methods has been demonstrated through extensive comparisons. Furthermore, we have developed a user-friendly script-like tool that streamlines the determination of descriptors and pK$_a$ values, enhancing the accessibility and practicality of our model. This work represents a significant advancement in the field of pK$_a$ prediction and holds great potential for applications in drug discovery, supramolecular chemistry, and other related disciplines. Through further optimization and refinement, the model can extend its predictive capabilities to diverse chemical systems beyond nitrogenous compounds.

## Methods

Prediction of pK$_a$ values of nitrogenous compounds using QSAR approach requires the calculation of relevant descriptors related to basicity or acidity. Chemical descriptors can be local or global, depending on whether they are related to a specific atom or group of atoms within the molecule or to the molecule as a whole. However, obtaining a comprehensive understanding of the electronic structure of a molecule and its acidity requires an advanced computational approach able to provide a detailed picture of the molecular parameters and properties relevant for predicting pK$_a$ values. In this section, we describe a comprehensive methodology for predicting basic pK$_a$ values of nitrogenous compounds.

### Prediction of pK$_a$ values using chemical descriptors

We employed a multi-faceted approach, using DFT as a fundamental computational method to determine electronic properties. DFT is widely used in quantum chemistry, since it is regarded as a reliable approach to predict molecular properties and structures [20].

However, DFT alone may not provide a complete picture of the molecular electronic structure. Therefore, additional post-processing techniques were employed. CDFT [30] was used to obtain a broader perspective of the molecular electronic structure by making use of global and local descriptors based on the conceptualization of electron density. Moreover, MEDT [24] and quantitative analysis of the molecular surface [58] were used to obtain properties that account for the electronic distribution of the molecule.

To obtain a comprehensive set of chemical descriptors, a combination of DFT, CDFT, MEDT, and quantitative analyses of molecular surface was used. Subsequently, a predictive model for pK$_a$ values was developed, using a diverse set of 130 training compounds in aqueous solution at 25 $^{\circ }$C, which included cyclic amines (aromatic and non-aromatic) and aliphatic amines (primary, secondary, and tertiary).

The most relevant descriptors for the prediction of pK$_a$ values were identified using QSARINS software for statistical analysis [32, 33]. QSARINS is a powerful tool for identifying the molecular descriptors that contribute most significantly to the predictive power of the model. QSARINS uses iterative techniques to add or remove descriptors from a multivariable linear equation, based on their statistical significance, to create a set of models from which the most suited is selected. The performance of the selected model was evaluated by testing it on a set of 40 validation compounds, which were not part of the training set of 130 compounds. This set included pharmaceutical ingredients and dyes with known pK$_a$ values.

### Determination of selected descriptors for pK$_a$ prediction

This section outlines the methodology for calculating the parameters involved in determining the pK$_a$ values according to Eq. 2 in the main section. The parameters include deprotonation energy ($\Delta$E), HOMO–LUMO deprotonation gap ($\Delta$HL$_{Gap}$), Mulliken electronegativity ($\chi _M$), the percentage of nonpolar surface area ($\%$NPSA), and the change in average local ionization energy at the nitrogen atom ($\Delta$ALIE$_N$).

To calculate these parameters swiftly and reliably, DFT calculations were run on optimized geometries under vacuum conditions. The B97-3c low-cost Density Functional Method [16] and the ORCA software package (Program Version 5.0.3) [66] were employed for this purpose. A more detailed description of the methodology used for each parameter is provided in the following sections.

#### Energy of deprotonation ($\Delta$E)

The electronic structure optimization and energy calculation for determining the energy of deprotonation ($\Delta$E) were carried out using the ORCA software package [66] with the B97-3c low-cost functional to obtain the most stable conformation at a local minimum of the base and conjugate acid, swiftly and reliably [16]. Protocol tightSCF was employed to ensure convergence. The total energy of the molecule was calculated, taking into account the Becke–Johnson dispersion damping (DFT-D3BJ) [34, 35] and short-range basis incompleteness SRB correction of the basis set [16, 87]. The energy change during the deprotonation process of the conjugate acid was calculated as the difference between the total energy of the base and the total energy of the conjugate acid:

3 $$\begin{aligned} \Delta E = \text {Total energy of base} - \text{Total energy of conjugate acid} \end{aligned}$$

It is important to note that this methodology requires the base to have a net charge of zero and the conjugate acid tohave a net charge of + 1. If the base is an ion, it should be neutralized with its respective counterion (Cl$^-$ or Na$^+$).

#### HOMO–LUMO gap of deprotonation ($\Delta$HL$_{Gap}$)

The HOMO–LUMO gap (HL$_{Gap}$) is a crucial parameter for characterizing the electronic properties of a system. The HOMO and LUMO energies were automatically determined at the end of the electronic structure optimization in the previous step. Specifically, the HOMO and LUMO energies were obtained from the eigenvalues of the highest occupied and lowest unoccupied molecular orbitals, respectively. Subsequently, the HOMO–LUMO gap energy was calculated by subtracting the LUMO energy from the HOMO energy.

4 $$\begin{aligned} HL_{Gap} = \epsilon _{LUMO} - \epsilon _{HOMO} \end{aligned}$$

Thus, the variation of the HL$_{Gap}$ in the deprotonation process of the conjugate acid was calculated as:

5 $$\begin{aligned} \Delta HL_{Gap} = HL_{Gap} \text { of base } - HL_{Gap} \text { of conjugate acid} \end{aligned}$$

#### Mulliken electronegativity ($\chi _M$)

Quantifies the ability of the base to donate a pair of electrons and accept a proton. $\chi _M$ was determined by Eq. 6 [69, 72]

Thus, the variation of the HL$_{Gap}$ in the deprotonation process of the conjugate acid was calculated as:

6 $$\begin{aligned} \chi _M = \frac{1}{2} (VIP + VEA) \end{aligned}$$

Were VIP and VEA are the vertical ionization potential and vertical electron affinity of the base, respectively. The energy of the neutral molecule was calculated by single point from optimized base using the same quantum chemistry software package and level of theory employed in the preceding calculations. Next, the energy of the cation (N − 1) was obtained by removing an electron from the neutral molecule (N) using the same software package and level of theory, setting the charge of the molecule to + 1 in the input file. The VIP was calculated as the energy difference between the cation (E$_{N-1}$) and the neutral molecule (E$_N$) [17],

7 $$\begin{aligned} VIP = E_{N-1} - E_N \end{aligned}$$

The energy of the anion (N + 1) was obtained by adding an electron to the neutral molecule (N) using the same software package and level of theory, setting the charge of the molecule to − 1 in the input file. The VEA was calculated as the energy difference between the neutral molecule (E$_N$) and the anion (E$_{N+1}$) [17],

8 $$\begin{aligned} VEA = E_N - E_{N+1} \end{aligned}$$

#### Nonpolar surface area percentage ($\%$NPSA)

The percentage of nonpolar surface area of the base was calculated using Multiwfn version 3.8 [57], a software package for post-processing wavefunction analysis, with an improved Marching Tetrahedra algorithm [58]. The molecular structure of the base was loaded into the software in Gaussian Binary Wavefunction format (.gbw) and analyzed using the “quantitative analysis of molecular surface” function with electrostatic potential (ESP) as the mapped function. The analysis was conducted under default settings, with an electron density contour value of 0.00100 used to define the isovalue of the electron density surface. The grid point spacing of 0.250000 was selected for generating the molecular surface, and the ratio of van der Waals radius was set to 1.7000 to extend the spatial region of cubic grids, which determines the size of the molecular surface by expanding the van der Waals radii of the atoms in the molecule.

#### Change in average local ionization energy at nitrogen atom ($\Delta$ALIE$_N$)

The reactivity of the acid–base reaction center was investigated using the concept of Average Local Ionization Energy (ALIE) [71, 85]. To compute the ALIE values for the nitrogen atoms in both the base and conjugate acid, Eq. 9 was employed.

9 $$\begin{aligned} ALIE_N = \sum \limits _{i}\rho _i (N) \frac{|\epsilon _i|}{\rho (N)} \end{aligned}$$

where $\rho _i$(N) denotes the density of the i-th orbital of the nitrogen atom, $\epsilon _i$ refers to the corresponding orbital energy, and $\rho$(N) denotes the total electron density on the nitrogen atom. The calculations were performed using Multiwfn software (version 3.8) [57] by importing the optimized molecular structures in.gbw format.

The difference between the ALIE$_N$ of the conjugate acid and the ALIE$_N$ of the base was calculated to determine $\Delta$ALIE$_N$, which provides a quantitative measure of the change in the electronic structure and potential energy of the nitrogen atom upon the acid–base reaction. The parameter $\Delta$ALIE$_N$ was calculated using the following equation:

10 $$\begin{aligned} \Delta ALIE_N = ALIE_N \text { of base }- ALIE_N \text { of acid} \end{aligned}$$

## Supplementary Information

**Additional file 1.** Optimized structures of the investigated compounds, equations, tables and additional figures.

## Data Availability

We have made the inputs used in this study publicly available, as well as a script that can estimate the basic pKa based on the structure of the base and the conjugate acid. You can access the inputs and the script at the following GitHub repository: https://github.com/Jacksonalcazar/Basic-pKa-Estimation-Nitrogen-Compounds.

## Footnotes

1 For the full equation, complete with error coefficients, please refer to Additional file 1: Equation S1.
