# Supplementary material for: Reliable and accurate prediction of basic pKa values in nitrogen compounds: the pKa shift in supramolecular systems as a case study
Source: J Cheminform. 2023 Sep 28;15:90. doi: 10.1186/s13321-023-00763-3 (PMC10540475; doi:10.1186/s13321-023-00763-3)
Supplement: Supplementary file 1 — Additional file 1. Optimized structures of the investigated compounds, equations, tables and additional figures. [file 13321_2023_763_MOESM1_ESM.zip › Supporting_Information.docx]

Supporting Information

**Reliable and accurate prediction of basic pK_a_ values in nitrogen compounds: The pK_a_ shift in supramolecular systems as a case study**

Jackson J. Alcázar,*^a^ Alessandra C. Misad Saide,^b^ and Paola R. Campodónico^a^

^a^ Centro de Química Médica, Facultad de Medicina Clínica Alemana, Universidad del Desarrollo, Santiago 7780272, Chile

^b^ Non-institutional affiliation

^*^ Author corresponding: jackson.alcazar@udd.cl

Table of Contents

[**Table S1.** Descriptors selected and their individual correlation with experimental pK_a_ in the trained set.	1](#_Toc138848111)

[**Table S2.** Correlation matrix of selected descriptors in the trained set.	1](#_Toc138848112)

[**Table S3.** Experimental and calculated pK_a_ values of nitrogen compounds in the trained set at 25 °C in aqueous solution.	1](#_Toc138848113)

[**Table S4.** Descriptors selected in the trained set and their respective values.	3](#_Toc138848114)

[**Table S5.** IUPAC name of some nitrogen compounds.	4](#_Toc138848115)

[**Figure S1.** Comparison of calculated pK_a_ values versus experimental pK_a_ values using First Principles calculations.	5](#_Toc138848116)

**pK_a_** = 0.1074(±0.0052)**ΔE** – 0.1422(±0.0290)**ΔHL_Gap_** – 0.9132(±0.0711)***χ*_M_** + 0.0151(±0.0026)**%NPSA** – 1.4887(±0.0486)**ΔALIE_N_** + 3.0608(±0.0817)**BaseT** – 30.7139(±1.3287)

Eq. S1

# **Table S1.** Descriptors selected and their individual correlation with experimental pK_a_ in the trained set.

| Correlation | ΔE | ΔHL_Gap_ | χ_M_ | %NPSA | ΔALIE_N_ | BaseT |
| --- | --- | --- | --- | --- | --- | --- |
| r | 0.6517 | -0.4356 | -0.5240 | 0.7514 | -0.3501 | 0.9168 |

According to data in Table S1, the %NPSA descriptor demonstrated one of the most significant individual correlations with the experimental pKa values (r = 0.7514). This intriguing finding emphasizes the relevance of %NPSA in predicting and understanding the variation in pKa values. The strong correlation of descriptor suggests that hydrophobicity, as measured by %NPSA, plays a crucial role in determining the acidity of the nitrogen-containing compounds. Consequently, this descriptor could be valuable in future studies aiming to predict pKa values.

# **Table S2.** Correlation matrix of selected descriptors in the trained set.

|  | ΔE | ΔHL_Gap_ | χ_M_ | %NPSA | ΔALIE_N_ | BaseT |
| --- | --- | --- | --- | --- | --- | --- |
| ΔE | 1 |  |  |  |  |  |
| ΔHL_Gap_ | -0.2962 | 1 |  |  |  |  |
| χ_M_ | -0.2619 | 0.3834 | 1 |  |  |  |
| %NPSA | 0.7744 | -0.541 | -0.4616 | 1 |  |  |
| ΔALIE_N_ | 0.2915 | -0.1011 | -0.0231 | 0.0535 | 1 |  |
| BaseT | 0.5130 | -0.3969 | -0.4591 | 0.5816 | -0.2472 | 1 |

# **Table S3.** Experimental and calculated pK_a_ values of nitrogen compounds in the trained set at 25 °C in aqueous solution.

| ID | Nitrogenous compounds | pK_a_ (exp)^a^ | pK_a_ (calc)^b^ | \|Error\| | pK_a_ (calc)^c^ | \|Error\| |
| --- | --- | --- | --- | --- | --- | --- |
| 1 | Quinuclidine | 11.30^d^ | 11.05 | 0.25 | 10.90 | 0.40 |
| 2 | Piperidine | 11.22 | 10.90 | 0.32 | 10.40 | 0.82 |
| 3 | Pyrrolidine | 11.10 | 10.77 | 0.33 | 10.40 | 0.70 |
| 4 | Diisopropylamine | 11.05 | 10.59 | 0.46 | 10.72 | 0.33 |
| 5 | (*R,S*)-Di-*S*-butylamine | 11.01 | 10.62 | 0.39 | 10.93 | 0.08 |
| 6 | Dipropylamine | 11.00 | 10.61 | 0.39 | 10.77 | 0.23 |
| 7 | Diethylamine | 10.98 | 10.79 | 0.19 | 10.58 | 0.40 |
| 8 | Tributylamine | 10.89 | 10.41 | 0.48 | 10.82 | 0.07 |
| 9 | Dimethylamine | 10.73 | 11.13 | 0.40 | 10.01 | 0.72 |
| 10 | Ethanamine | 10.70 | 10.58 | 0.12 | 10.23 | 0.47 |
| 11 | Heptan-1-amine | 10.70 | 10.49 | 0.21 | 10.21 | 0.49 |
| 12 | Octan-1-amine | 10.65 | 10.48 | 0.17 | 10.21 | 0.44 |
| 13 | Triethylamine | 10.65 | 10.63 | 0.02 | 10.21 | 0.44 |
| 14 | Tripropylamine | 10.65 | 10.46 | 0.19 | 10.82 | 0.17 |
| 15 | Cyclohexanamine | 10.64 | 10.41 | 0.23 | 10.45 | 0.19 |
| 16 | Propan-2-amine | 10.63 | 10.74 | 0.11 | 10.43 | 0.20 |
| 17 | Pentan-1-amine | 10.60 | 10.52 | 0.08 | 10.21 | 0.39 |
| 18 | Hexan-1-amine | 10.60 | 10.50 | 0.10 | 10.21 | 0.39 |
| 19 | Butan-1-amine | 10.59 | 10.54 | 0.05 | 10.21 | 0.38 |
| 20 | (*S*)-Butan-2-amine | 10.56 | 10.48 | 0.08 | 10.44 | 0.12 |
| 21 | Butan-2-amine | 10.56 | 10.46 | 0.10 | 10.44 | 0.12 |
| 22 | 2-Methylpropan-1-amine | 10.55 | 10.49 | 0.06 | 10.24 | 0.31 |
| 23 | 2-Methylpropan-2-amine | 10.55 | 10.37 | 0.18 | 10.65 | 0.10 |
| 24 | Propan-1-amine | 10.53 | 10.58 | 0.05 | 10.21 | 0.32 |
| 25 | *N,N*,2-Trimethylpropan-2-amine | 10.52 | 10.63 | 0.11 | 10.24 | 0.28 |
| 26 | Diisobutylamine | 10.50 | 10.48 | 0.02 | 11.03 | 0.53 |
| 27 | *N*-Methylcyclohexanamine | 10.49 | 10.48 | 0.01 | 10.70 | 0.21 |
| 28 | *N*-Methylpyrrolidine | 10.46 | 10.50 | 0.04 | 10.08 | 0.38 |
| 29 | (*R*)*-N,N*-Dimethylpentan-2-amine | 10.40 | 10.35 | 0.05 | 10.07 | 0.33 |
| 30 | 1-Ethylpiperidine | 10.40 | 10.42 | 0.02 | 10.06 | 0.34 |
| 31 | *N^1^,N^2^*-Dimethylethane-1,2-diamine | 10.40 | 10.23 | 0.17 | 9.85 | 0.55 |
| 32 | *N,N*-Dimethylbutan-2-amine | 10.40 | 10.43 | 0.03 | 10.13 | 0.27 |
| 33 | *N,N*-Dimethylpropan-2-amine | 10.30 | 10.56 | 0.26 | 9.98 | 0.32 |
| 34 | *N*-Ethyl-*N*-methylethanamine | 10.29 | 10.60 | 0.31 | 9.97 | 0.32 |
| 35 | 2,2-Dimethylpropan-1-amine | 10.21 | 10.36 | 0.15 | 9.93 | 0.28 |
| 36 | *N*-Methylprop-2-en-1-amine | 10.11 | 9.96 | 0.15 | 9.80 | 0.31 |
| 37 | 3-Methoxypropan-1-amine | 10.10 | 9.95 | 0.15 | 10.09 | 0.01 |
| 38 | *N*-Cyclohexylethanolamine | 10.10 | 9.91 | 0.19 | 10.18 | 0.08 |
| 39 | 1-Methylpiperidine | 10.08 | 10.46 | 0.38 | 9.81 | 0.27 |
| 40 | *N,N*-Dimethylbutan-1-amine | 10.02 | 10.39 | 0.37 | 9.79 | 0.23 |
| 41 | *N,N*-Dimethylpropan-1-amine | 9.99 | 10.50 | 0.51 | 9.87 | 0.12 |
| 42 | Ethane-1,2-diamine | 9.98 | 9.50 | 0.48 | 9.69 | 0.29 |
| 43 | (*R*)-*N,N*,2-Trimethylbutan-1-amine | 9.91 | 10.51 | 0.60 | 10.01 | 0.10 |
| 44 | 1-Methylazepane | 9.91 | 10.42 | 0.51 | 10.18 | 0.27 |
| 45 | *N,N*,2-Trimethylpropan-1-amine | 9.91 | 10.20 | 0.29 | 10.05 | 0.14 |
| 46 | (*S*)-N-Methyl-1-phenylpropan-2-amine | 9.87 | 9.72 | 0.15 | 10.21 | 0.34 |
| 47 | *N*-Methylethanolamine | 9.85 | 9.73 | 0.12 | 9.81 | 0.04 |
| 48 | Piperazine | 9.73 | 10.35 | 0.62 | 9.56 | 0.17 |
| 49 | *N*-Benzylethanamine | 9.68 | 9.34 | 0.34 | 9.49 | 0.19 |
| 50 | 1-Allylpiperidine | 9.68 | 9.52 | 0.16 | 9.41 | 0.27 |
| 51 | *N*-Methyl-1-phenylmethanamine | 9.58 | 9.37 | 0.21 | 9.41 | 0.17 |
| 52 | 2-(3,4,5-Trimethoxyphenyl)ethan-1-amine | 9.56 | 10.08 | 0.52 | 9.32 | 0.24 |
| 53 | Ethanolamine | 9.50 | 9.21 | 0.29 | 9.55 | 0.05 |
| 54 | Prop-2-en-1-amine | 9.49 | 9.74 | 0.25 | 9.66 | 0.17 |
| 55 | *N*-Benzyl-N-ethylethanamine | 9.48 | 9.11 | 0.37 | 9.46 | 0.02 |
| 56 | 2-Methylbenzylamine | 9.44 | 9.27 | 0.17 | 9.31 | 0.13 |
| 57 | (*R*)-Venlafaxine | 9.40 | 9.87 | 0.47 | 8.76 | 0.64 |
| 58 | Phenylmethanamine | 9.34 | 9.24 | 0.10 | 9.30 | 0.04 |
| 59 | Diallylamine | 9.29 | 9.10 | 0.19 | 9.16 | 0.13 |
| 60 | *N,N*-Dimethyl-1-phenylmethanamine | 8.93 | 9.12 | 0.19 | 8.90 | 0.03 |
| 61 | *N*-Allyl-*N*-methylprop-2-en-1-amine | 8.79 | 8.77 | 0.02 | 8.62 | 0.17 |
| 62 | Cysteamine | 8.35 | 8.62 | 0.27 | 10.13 | 1.78 |
| 63 | *N*-Benzoylpiperazine | 7.78 | 8.31 | 0.53 | 7.82 | 0.04 |
| 64 | Triethanolamine | 7.77 | 8.03 | 0.26 | 7.79 | 0.02 |
| 65 | 3-Aminopropanenitrile | 7.70 | 7.25 | 0.45 | 7.85 | 0.15 |
| 66 | (*S*)-2-(Diethylamino)Propanenitrile | 7.60 | 7.40 | 0.20 | 6.22 | 1.38 |
| 67 | 2,2-Difluoroethan-1-amine | 7.52 | 7.94 | 0.42 | 6.54 | 0.98 |
| 68 | 1-Tosylpiperazine | 7.39 | 7.82 | 0.43 | 7.16 | 0.23 |
| 69 | 3-(Dimethylamino)Propanenitrile | 7.00 | 7.29 | 0.29 | 6.92 | 0.08 |
| 70 | (*R*)-3-(Methyl(1-phenylpropan-2-yl)amino)Propanenitrile | 6.95 | 6.92 | 0.03 | 7.39 | 0.44 |
| 71 | 4-Ethylpyridine | 6.02 | 5.58 | 0.44 | 5.76 | 0.26 |
| 72 | 4-(Tert-butyl)Pyridine | 5.99 | 5.70 | 0.29 | 5.57 | 0.42 |
| 73 | Hydroxylamine | 5.97 | 5.77 | 0.20 | 3.94 | 2.03 |
| 74 | 2-Ethylpyridine | 5.97 | 5.62 | 0.35 | 5.64 | 0.33 |
| 75 | *N*-Methylhydroxylamine | 5.96 | 6.37 | 0.41 | 4.89 | 1.07 |
| 76 | 3-(Tert-butyl)pyridine | 5.82 | 5.86 | 0.04 | 5.47 | 0.35 |
| 77 | 2-(Tert-butyl)pyridine | 5.76 | 5.76 | 0.00 | 5.29 | 0.47 |
| 78 | 3-Ethylpyridine | 5.70 | 5.61 | 0.09 | 5.57 | 0.13 |
| 79 | Methoxyamine | 5.59 | 6.24 | 0.65 | 4.42 | 1.17 |
| 80 | 2-Methyl-4,5-dihydrooxazole | 5.50 | 6.13 | 0.63 | 4.28 | 1.22 |
| 81 | 4-Methoxyaniline | 5.29 | 5.02 | 0.27 | 5.11 | 0.18 |
| 82 | 4-Ethoxyaniline | 5.25 | 5.15 | 0.10 | 5.13 | 0.12 |
| 83 | Pyridine | 5.24 | 5.26 | 0.02 | 5.12 | 0.12 |
| 84 | 3,3'-Azanediyldipropanenitrile | 5.20 | 5.23 | 0.03 | 5.66 | 0.46 |
| 85 | Benzo[*f*]quinoline | 5.15 | 4.82 | 0.33 | 4.64 | 0.51 |
| 86 | Isoquinoline | 5.14 | 4.98 | 0.16 | 5.26 | 0.12 |
| 87 | 4-Methylaniline | 5.07 | 5.01 | 0.06 | 4.99 | 0.08 |
| 88 | Benzo[*g*]quinoline | 5.05 | 4.75 | 0.30 | 4.69 | 0.36 |
| 89 | Benzene-1,3-diamine | 4.88 | 4.79 | 0.09 | 5.48 | 0.60 |
| 90 | 3-Hydroxypyridine | 4.86 | 5.09 | 0.23 | 4.86 | 0.00 |
| 91 | Quinoline | 4.85 | 4.80 | 0.05 | 4.90 | 0.05 |
| 92 | Benzo[*a*]acridine | 4.70 | 4.63 | 0.07 | 5.97 | 1.27 |
| 93 | Aniline | 4.70 | 4.53 | 0.17 | 4.64 | 0.06 |
| 94 | 3-Methylaniline | 4.67 | 4.77 | 0.10 | 5.36 | 0.69 |
| 95 | 3-(Trimethylsilyl)aniline | 4.64 | 4.61 | 0.03 | 4.50 | 0.14 |
| 96 | 4-Fluoroaniline | 4.52 | 4.01 | 0.51 | 4.40 | 0.12 |
| 97 | Benzo[*b*]acridine | 4.52 | 4.63 | 0.11 | 5.98 | 1.46 |
| 98 | 3-Methoxyaniline | 4.49 | 4.52 | 0.03 | 4.01 | 0.48 |
| 99 | Benzene-1,2-diamine | 4.47 | 4.39 | 0.08 | 4.74 | 0.27 |
| 100 | 4-(Methylthio)Aniline | 4.40 | 3.83 | 0.57 | 4.55 | 0.15 |
| 101 | 2-Phenyl-4,5-dihydrooxazole | 4.40 | 4.86 | 0.46 | 4.15 | 0.25 |
| 102 | 2-Methylaniline | 4.38 | 4.70 | 0.32 | 4.48 | 0.10 |
| 103 | 4-(Trimethylsilyl)Aniline | 4.36 | 4.57 | 0.21 | 4.51 | 0.15 |
| 104 | 4-Phenylaniline | 4.27 | 3.92 | 0.35 | 4.28 | 0.01 |
| 105 | Benzo[*h]*quinoline | 4.25 | 4.58 | 0.33 | 3.95 | 0.30 |
| 106 | 3-Phenylaniline | 4.18 | 3.98 | 0.20 | 4.34 | 0.16 |
| 107 | 3-Aminophenol | 4.17 | 4.28 | 0.11 | 4.02 | 0.15 |
| 108 | 3-Ethoxyaniline | 4.17 | 4.73 | 0.56 | 4.03 | 0.14 |
| 109 | 3-(Methylthio)Aniline | 4.05 | 4.17 | 0.12 | 4.09 | 0.04 |
| 110 | 4-Bromoaniline | 3.91 | 3.37 | 0.54 | 4.75 | 0.84 |
| 111 | 4-Chloroaniline | 3.81 | 3.49 | 0.32 | 4.09 | 0.28 |
| 112 | 2-Phenylaniline | 3.78 | 3.99 | 0.21 | 4.08 | 0.30 |
| 113 | Methyl 3-aminobenzoate | 3.56 | 3.21 | 0.35 | 3.65 | 0.09 |
| 114 | 3-Bromoaniline | 3.51 | 3.16 | 0.35 | 3.91 | 0.40 |
| 115 | 3-Fluoroaniline | 3.38 | 3.56 | 0.18 | 3.22 | 0.16 |
| 116 | 3-Chloroaniline | 3.32 | 3.27 | 0.05 | 3.47 | 0.15 |
| 117 | Quinazoline | 3.31 | 3.71 | 0.40 | 3.28 | 0.03 |
| 118 | 1,4-Dihydroquinazoline | 3.31 | 3.67 | 0.36 | 6.93 | 3.62 |
| 119 | 4-Aminobenzenaminium chloride | 3.29 | 3.30 | 0.01 | 3.04 | 0.25 |
| 120 | 3-Aminobenzoic acid | 3.05 | 2.87 | 0.18 | 3.27 | 0.22 |
| 121 | (*S*)-2-Phenyl-4,5-dihydrooxazole-4-carboxamide | 2.90 | 3.45 | 0.55 | 0.95 | 1.95 |
| 122 | 3-(Methylsulfonyl)Aniline | 2.68 | 2.04 | 0.64 | 2.37 | 0.31 |
| 123 | 3-Aminobenzenaminium chloride | 2.65 | 2.95 | 0.30 | 2.73 | 0.08 |
| 124 | 4-(Trifluoromethyl)Aniline | 2.57 | 2.47 | 0.10 | 3.60 | 1.03 |
| 125 | 4-Aminobenzoic acid | 2.32 | 2.08 | 0.24 | 2.69 | 0.37 |
| 126 | Methyl 4-aminobenzoate | 2.30 | 2.53 | 0.23 | 2.74 | 0.44 |
| 127 | 2-Aminobenzoic acid | 2.14 | 2.21 | 0.07 | 1.95 | 0.19 |
| 128 | 4-(Methylsulfonyl)Aniline | 1.48 | 1.77 | 0.29 | 1.85 | 0.37 |
| 129 | 2-Aminobenzenaminium chloride | 1.30 | 1.34 | 0.04 | 0.56 | 0.74 |
| 130 | 4-Nitroaniline | 0.11 | 0.60 | 0.49 | 2.13 | 2.02 |
|  |  |  | **MAE:** | 0.24 | **MAE:** | 0.40 |

^a^ Experimental data taken from Ref. [99]. ^b^ Calculated by Eq. 2. RMSE = 0.30. ^c^ Calculated by Chemaxon software. RMSE = 0.63. ^d^ Experimental data were taken from Ref. [100].

# **Table S4.** Descriptors selected in the trained set and their respective values.

| ID | Nitrogenous compounds | ΔE  (kcal/mol) | ΔHL_Gap_  (eV) | χ_M_  (eV) | %NPSA | ΔALIE_N_  (eV) | BaseT |
| --- | --- | --- | --- | --- | --- | --- | --- |
| 1 | Quinuclidine | 247.53 | -2.36 | 2.62 | 92.86 | -8.58 | 1 |
| 2 | Piperidine | 242.58 | -1.91 | 2.77 | 87.42 | -9.02 | 1 |
| 3 | Pyrrolidine | 240.25 | -2.02 | 2.91 | 84.57 | -9.21 | 1 |
| 4 | Diisopropylamine | 246.17 | -2.94 | 2.82 | 89.19 | -8.47 | 1 |
| 5 | (*R,S*)-Di-*S*-butylamine | 250.27 | -2.32 | 2.80 | 91.59 | -8.22 | 1 |
| 6 | Dipropylamine | 243.44 | -1.84 | 2.87 | 89.39 | -8.82 | 1 |
| 7 | Diethylamine | 241.16 | -2.84 | 2.80 | 85.21 | -9.00 | 1 |
| 8 | Tributylamine | 252.03 | -2.14 | 2.55 | 97.50 | -7.76 | 1 |
| 9 | Dimethylamine | 235.04 | -4.12 | 2.91 | 79.35 | -9.68 | 1 |
| 10 | Ethanamine | 232.23 | -2.36 | 3.23 | 64.81 | -10.02 | 1 |
| 11 | Heptan-1-amine | 234.99 | 1.27 | 3.01 | 84.40 | -9.77 | 1 |
| 12 | Octan-1-amine | 235.08 | 1.59 | 2.98 | 85.89 | -9.76 | 1 |
| 13 | Triethylamine | 247.48 | -3.38 | 2.53 | 94.54 | -8.13 | 1 |
| 14 | Tripropylamine | 250.29 | -2.76 | 2.56 | 96.89 | -7.87 | 1 |
| 15 | Cyclohexanamine | 238.22 | -0.36 | 3.13 | 81.55 | -9.44 | 1 |
| 16 | Propan-2-amine | 236.80 | -2.35 | 3.16 | 73.45 | -9.67 | 1 |
| 17 | Pentan-1-amine | 234.61 | 0.38 | 3.05 | 80.17 | -9.80 | 1 |
| 18 | Hexan-1-amine | 234.84 | 0.88 | 3.02 | 82.54 | -9.78 | 1 |
| 19 | Butan-1-amine | 234.19 | -0.29 | 3.09 | 77.13 | -9.84 | 1 |
| 20 | (*S*)-Butan-2-amine | 236.67 | -1.28 | 3.17 | 76.32 | -9.59 | 1 |
| 21 | Butan-2-amine | 236.79 | -1.70 | 3.20 | 79.12 | -9.51 | 1 |
| 22 | 2-Methylpropan-1-amine | 234.58 | -1.19 | 3.12 | 76.54 | -9.72 | 1 |
| 23 | 2-Methylpropan-2-amine | 237.95 | -2.32 | 3.30 | 74.87 | -9.41 | 1 |
| 24 | Propan-1-amine | 233.42 | -1.16 | 3.12 | 72.59 | -9.91 | 1 |
| 25 | *N,N*,2-Trimethylpropan-2-amine | 246.92 | -3.13 | 2.59 | 95.45 | -8.22 | 1 |
| 26 | Diisobutylamine | 245.67 | -1.91 | 2.92 | 92.50 | -8.56 | 1 |
| 27 | *N*-Methylcyclohexanamine | 243.57 | -0.94 | 2.83 | 89.10 | -8.79 | 1 |
| 28 | *N*-Methylpyrrolidine | 242.91 | -2.30 | 2.68 | 93.88 | -8.57 | 1 |
| 29 | (*R*)*-N,N*-Dimethylpentan-2-amine | 246.19 | -1.57 | 2.65 | 95.13 | -8.28 | 1 |
| 30 | 1-Ethylpiperidine | 247.17 | -2.42 | 2.62 | 95.82 | -8.14 | 1 |
| 31 | *N^1^,N^2^*-Dimethylethane-1,2-diamine | 239.66 | 1.74 | 2.55 | 72.11 | -9.15 | 1 |
| 32 | *N,N*-Dimethylbutan-2-amine | 245.44 | -2.54 | 2.67 | 95.13 | -8.31 | 1 |
| 33 | *N,N*-Dimethylpropan-2-amine | 244.19 | -3.01 | 2.65 | 93.92 | -8.43 | 1 |
| 34 | *N*-Ethyl-*N*-methylethanamine | 244.46 | -3.21 | 2.66 | 94.62 | -8.42 | 1 |
| 35 | 2,2-Dimethylpropan-1-amine | 235.53 | -1.28 | 3.16 | 78.67 | -9.55 | 1 |
| 36 | *N*-Methylprop-2-en-1-amine | 239.50 | -1.12 | 3.10 | 71.52 | -9.05 | 1 |
| 37 | 3-Methoxypropan-1-amine | 232.48 | 2.35 | 2.91 | 65.31 | -9.83 | 1 |
| 38 | *N*-Cyclohexylethanolamine | 243.84 | -0.23 | 2.83 | 77.92 | -8.56 | 1 |
| 39 | 1-Methylpiperidine | 244.48 | -2.28 | 2.65 | 94.92 | -8.40 | 1 |
| 40 | *N,N*-Dimethylbutan-1-amine | 243.35 | -1.31 | 2.69 | 95.08 | -8.56 | 1 |
| 41 | *N,N*-Dimethylpropan-1-amine | 242.66 | -2.10 | 2.69 | 94.46 | -8.61 | 1 |
| 42 | Ethane-1,2-diamine | 230.57 | 1.53 | 2.76 | 34.14 | -9.82 | 1 |
| 43 | (*R*)-*N,N*,2-Trimethylbutan-1-amine | 245.35 | -1.35 | 2.68 | 95.21 | -8.48 | 1 |
| 44 | 1-Methylazepane | 246.14 | -1.79 | 2.65 | 95.60 | -8.29 | 1 |
| 45 | *N,N*,2-Trimethylpropan-1-amine | 242.20 | -2.43 | 2.51 | 95.72 | -8.29 | 1 |
| 46 | (*S*)-N-Methyl-1-phenylpropan-2-amine | 242.68 | -0.36 | 3.05 | 71.82 | -8.70 | 1 |
| 47 | *N*-Methylethanolamine | 235.34 | 0.27 | 2.92 | 60.98 | -9.33 | 1 |
| 48 | Piperazine | 240.69 | 0.52 | 2.60 | 69.47 | -9.11 | 1 |
| 49 | *N*-Benzylethanamine | 244.09 | -0.67 | 3.18 | 73.87 | -8.37 | 1 |
| 50 | 1-Allylpiperidine | 246.40 | -1.34 | 2.96 | 92.32 | -7.95 | 1 |
| 51 | *N*-Methyl-1-phenylmethanamine | 241.50 | -0.61 | 3.19 | 70.24 | -8.63 | 1 |
| 52 | 2-(3,4,5-Trimethoxyphenyl)ethan-1-amine | 239.17 | 1.95 | 2.76 | 52.96 | -9.43 | 1 |
| 53 | Ethanolamine | 228.61 | 0.90 | 3.29 | 35.04 | -10.02 | 1 |
| 54 | Prop-2-en-1-amine | 230.14 | -0.55 | 3.38 | 59.75 | -9.93 | 1 |
| 55 | *N*-Benzyl-N-ethylethanamine | 249.24 | -0.94 | 3.01 | 82.68 | -7.63 | 1 |
| 56 | 2-Methylbenzylamine | 236.42 | 0.36 | 3.31 | 63.07 | -9.17 | 1 |
| 57 | (*R*)-Venlafaxine | 258.18 | -0.06 | 2.75 | 69.07 | -7.56 | 1 |
| 58 | Phenylmethanamine | 235.48 | -0.20 | 3.35 | 51.38 | -9.31 | 1 |
| 59 | Diallylamine | 241.81 | -0.85 | 3.38 | 73.02 | -8.49 | 1 |
| 60 | *N,N*-Dimethyl-1-phenylmethanamine | 244.18 | -0.86 | 3.11 | 77.20 | -8.12 | 1 |
| 61 | *N*-Allyl-*N*-methylprop-2-en-1-amine | 243.18 | -1.08 | 3.34 | 85.47 | -8.00 | 1 |
| 62 | Cysteamine | 226.78 | 1.51 | 3.23 | 34.61 | -9.77 | 1 |
| 63 | *N*-Benzoylpiperazine | 236.86 | 0.31 | 3.18 | 48.95 | -8.55 | 1 |
| 64 | Triethanolamine | 239.45 | -0.65 | 2.96 | 52.07 | -7.91 | 1 |
| 65 | 3-Aminopropanenitrile | 217.55 | -0.24 | 3.78 | 20.65 | -9.84 | 1 |
| 66 | (*S*)-2-(Diethylamino)Propanenitrile | 233.36 | -1.84 | 3.15 | 54.28 | -7.91 | 1 |
| 67 | 2,2-Difluoroethan-1-amine | 218.87 | -0.93 | 3.60 | 25.70 | -9.97 | 1 |
| 68 | 1-Tosylpiperazine | 235.89 | -0.52 | 3.69 | 44.06 | -8.57 | 1 |
| 69 | 3-(Dimethylamino)Propanenitrile | 228.51 | -1.37 | 3.29 | 25.24 | -8.62 | 1 |
| 70 | (*R*)-3-(Methyl(1-phenylpropan-2-yl)amino)Propanenitrile | 235.29 | -0.53 | 3.22 | 41.68 | -7.74 | 1 |
| 71 | 4-Ethylpyridine | 240.08 | -0.33 | 3.94 | 57.29 | -8.86 | 0 |
| 72 | 4-(Tert-butyl)Pyridine | 241.78 | -0.02 | 3.87 | 63.98 | -8.74 | 0 |
| 73 | Hydroxylamine | 208.15 | -0.91 | 3.78 | 27.44 | -11.44 | 0 |
| 74 | 2-Ethylpyridine | 240.18 | -0.03 | 3.86 | 70.03 | -8.73 | 0 |
| 75 | *N*-Methylhydroxylamine | 217.74 | -1.49 | 3.33 | 50.75 | -10.58 | 0 |
| 76 | 3-(Tert-butyl)pyridine | 241.25 | 0.17 | 3.80 | 70.28 | -8.79 | 0 |
| 77 | 2-(Tert-butyl)pyridine | 243.12 | 0.00 | 3.73 | 75.46 | -8.48 | 0 |
| 78 | 3-Ethylpyridine | 239.00 | 0.09 | 3.87 | 61.91 | -8.91 | 0 |
| 79 | Methoxyamine | 215.69 | -0.05 | 3.50 | 55.61 | -10.84 | 0 |
| 80 | 2-Methyl-4,5-dihydrooxazole | 237.49 | 0.14 | 3.16 | 51.47 | -9.04 | 0 |
| 81 | 4-Methoxyaniline | 231.44 | -0.25 | 2.84 | 52.19 | -8.49 | 0 |
| 82 | 4-Ethoxyaniline | 232.14 | -0.21 | 2.81 | 57.78 | -8.46 | 0 |
| 83 | Pyridine | 234.60 | -0.30 | 4.05 | 52.77 | -9.16 | 0 |
| 84 | 3,3'-Azanediyldipropanenitrile | 215.03 | -0.57 | 4.02 | 16.12 | -8.82 | 1 |
| 85 | Benzo[*f*]quinoline | 244.70 | 0.96 | 3.90 | 58.76 | -8.10 | 0 |
| 86 | Isoquinoline | 241.59 | 0.48 | 4.04 | 58.37 | -8.48 | 0 |
| 87 | 4-Methylaniline | 226.43 | -0.90 | 2.75 | 58.18 | -8.67 | 0 |
| 88 | Benzo[*g*]quinoline | 245.12 | 0.66 | 3.95 | 57.01 | -8.05 | 0 |
| 89 | Benzene-1,3-diamine | 228.78 | 0.52 | 2.50 | 36.05 | -8.56 | 0 |
| 90 | 3-Hydroxypyridine | 234.68 | 0.64 | 3.79 | 51.38 | -8.98 | 0 |
| 91 | Quinoline | 241.03 | 0.55 | 4.09 | 55.99 | -8.46 | 0 |
| 92 | Benzo[*a*]acridine | 250.24 | 0.42 | 4.00 | 59.08 | -7.58 | 0 |
| 93 | Aniline | 223.55 | -1.00 | 2.87 | 48.92 | -8.72 | 0 |
| 94 | 3-Methylaniline | 225.66 | -0.71 | 2.84 | 58.08 | -8.64 | 0 |
| 95 | 3-(Trimethylsilyl)aniline | 226.85 | -0.63 | 3.01 | 69.97 | -8.43 | 0 |
| 96 | 4-Fluoroaniline | 220.99 | -1.02 | 3.05 | 38.23 | -8.77 | 0 |
| 97 | Benzo[*b*]acridine | 252.12 | 0.30 | 3.97 | 58.00 | -7.42 | 0 |
| 98 | 3-Methoxyaniline | 226.83 | 0.22 | 2.71 | 40.95 | -8.57 | 0 |
| 99 | Benzene-1,2-diamine | 230.24 | -0.98 | 2.44 | 42.11 | -7.94 | 0 |
| 100 | 4-(Methylthio)Aniline | 226.56 | 0.72 | 2.97 | 50.35 | -8.24 | 0 |
| 101 | 2-Phenyl-4,5-dihydrooxazole | 242.42 | 0.30 | 3.76 | 49.76 | -8.24 | 0 |
| 102 | 2-Methylaniline | 225.34 | -0.76 | 2.78 | 55.64 | -8.60 | 0 |
| 103 | 4-(Trimethylsilyl)Aniline | 226.02 | -0.29 | 2.81 | 70.69 | -8.37 | 0 |
| 104 | 4-Phenylaniline | 227.68 | -0.05 | 3.08 | 45.74 | -8.26 | 0 |
| 105 | Benzo[*h]*quinoline | 242.48 | 0.87 | 3.84 | 54.97 | -8.09 | 0 |
| 106 | 3-Phenylaniline | 228.30 | -0.20 | 3.34 | 45.56 | -8.40 | 0 |
| 107 | 3-Aminophenol | 224.79 | 0.07 | 2.75 | 35.62 | -8.62 | 0 |
| 108 | 3-Ethoxyaniline | 227.77 | 0.26 | 2.68 | 50.60 | -8.53 | 0 |
| 109 | 3-(Methylthio)Aniline | 225.83 | 0.67 | 2.74 | 40.47 | -8.47 | 0 |
| 110 | 4-Bromoaniline | 219.63 | -0.18 | 3.07 | 38.39 | -8.52 | 0 |
| 111 | 4-Chloroaniline | 219.75 | -0.49 | 3.07 | 36.97 | -8.58 | 0 |
| 112 | 2-Phenylaniline | 230.01 | -0.84 | 3.20 | 58.95 | -8.00 | 0 |
| 113 | Methyl 3-aminobenzoate | 222.01 | -0.43 | 3.62 | 46.24 | -8.48 | 0 |
| 114 | 3-Bromoaniline | 218.99 | -0.01 | 3.19 | 40.49 | -8.50 | 0 |
| 115 | 3-Fluoroaniline | 218.89 | -0.45 | 3.05 | 39.62 | -8.66 | 0 |
| 116 | 3-Chloroaniline | 218.85 | -0.25 | 3.16 | 39.18 | -8.55 | 0 |
| 117 | Quinazoline | 232.90 | 0.10 | 4.44 | 54.42 | -8.50 | 0 |
| 118 | 1,4-Dihydroquinazoline | 233.20 | 0.23 | 4.44 | 54.42 | -8.46 | 0 |
| 119 | 4-Aminobenzenaminium chloride | 224.20 | -0.33 | 3.07 | 25.81 | -8.26 | 0 |
| 120 | 3-Aminobenzoic acid | 219.86 | -0.51 | 3.73 | 42.42 | -8.50 | 0 |
| 121 | (*S*)-2-Phenyl-4,5-dihydrooxazole-4-carboxamide | 234.48 | 0.78 | 3.85 | 55.96 | -7.90 | 0 |
| 122 | 3-(Methylsulfonyl)Aniline | 215.85 | -0.27 | 3.71 | 33.51 | -8.34 | 0 |
| 123 | 3-Aminobenzenaminium chloride | 223.01 | 0.15 | 3.12 | 26.23 | -8.18 | 0 |
| 124 | 4-(Trifluoromethyl)Aniline | 214.50 | -0.82 | 3.38 | 36.94 | -8.43 | 0 |
| 125 | 4-Aminobenzoic acid | 216.32 | 0.13 | 3.61 | 40.62 | -8.23 | 0 |
| 126 | Methyl 4-aminobenzoate | 218.65 | 0.22 | 3.49 | 47.58 | -8.23 | 0 |
| 127 | 2-Aminobenzoic acid | 222.09 | -1.07 | 3.66 | 46.92 | -7.76 | 0 |
| 128 | 4-(Methylsulfonyl)Aniline | 214.11 | 0.41 | 3.57 | 33.24 | -8.26 | 0 |
| 129 | 2-Aminobenzenaminium chloride | 226.05 | -0.02 | 3.39 | 32.40 | -6.97 | 0 |
| 130 | 4-Nitroaniline | 208.82 | -0.25 | 4.24 | 40.46 | -8.14 | 0 |

# **Table S5.** IUPAC name of some nitrogen compounds.

| ID | Nitrogenous compounds | IUPAC Name |
| --- | --- | --- |
| 162 | 7-Dimethylazacoumarin | 7-(dimethylamino)-3-[(E)-2-phenylethenyl]-1,4-benzoxazin-2-one |
| 163 | CHEMBL1689126 | (*S*)-2-(4-chloro-2-((3-methyl-4-(2-phenylacetyl)piperazin-1-yl)methyl)phenoxy)acetic acid |
| 164 | CHEMBL1689112 | 2-(2-((4-benzoylpiperazin-1-yl)methyl)-4-(trifluoromethyl)phenoxy)acetic acid |
| 165 | CHEMBL47529 | 2-(2-methyl-8-phenylmethoxyimidazo[1,2-a]pyridin-3-yl)acetonitrile |
| 166 | CHEMBL1349378 | 4-(hydroxymethyl)quinolin-8-ol |
| 167 | CHEMBL432733 | 2-amino-5-methyl-4-phenyl-1*H*-pyrimidin-6-one |
| 168 | CHEMBL271703 | (*R*)-2-((7-amino-2-((2,3-difluorobenzyl)thio)pteridin-4-yl)amino)propan-1-ol |
| 169 | CHEMBL191553 | *N*-[4-[[(4-phenoxy-1*H*-pyrazolo[4,3-c]pyridin-3-yl)amino]methyl]phenyl]acetamide |
| 170 | CHEMBL1405150 | *N*-((5-chloro-8-hydroxyquinolin-7-yl)(thiophen-2-yl)methyl)propionamide |

First Principles Calculations

${pK}_{a}(B)= {pK}_{a}\left( R \right)+ \frac{G_{gas}\left( B \right)-G_{gas}\left( R \right)-G_{gas}\left( {HB}^{+} \right)+G_{gas}\left( {HR}^{+} \right)+{\Delta G}_{sol}\left( B \right)-\Delta G_{sol}\left( R \right)-\Delta G_{sol}\left( {HB}^{+} \right)+{\Delta G}_{sol}\left( {HR}^{+} \right)}{RTln(10)}$	Eq. S2.

Here, R represents the reference species with a known pK_a_ value, B denote the base, G symbolize the Gibbs free energy, "gas" signifies the gas phase, "sol" indicates the solution phase, R represents the gas constant, T represents the temperature, and HR^+^ and HB^+^ refer to the conjugate acids of the bases B and R, respectively.

# **Figure S1.** Comparison of calculated pK_a_ values versus experimental pK_a_ values using the equation pK_a_ = 106.41pK_a_(B) - 749.49, based on a training set of 130 molecules. pK_a(ΔG)_ values were determined using Eq. S2.

To demonstrate the limitations of pK_a_ prediction using First Principles calculations with the same level of theory (B97-3C), we employed the popular thermodynamic cycle approach relative to a known pK_a_ reference compound (Eq. S2). The results are depicted in Figure S1. Upon comparing the predicted pK_a_ values with experimental data for the same training set as in the QSAR approach, we observed significant discrepancies. These disparities between the predicted and experimental pK_a_ values can be attributed to several factors.

Firstly, the thermodynamic cycle approach assumes that the reference compound (water, in this case) accurately represents the behavior of other compounds, regardless of their unique chemical properties and electronic structure. This oversimplification can lead to errors in pK_a_ prediction and underscores the dependence of the model on the reference system.

Moreover, the accuracy of the energetic contributions, such as gas-phase and solvation free energies, also influences the reliability of pK_a_ prediction. Even small errors or approximations in these energetic terms can propagate and substantially impact the final pK_a_ values.

# **References**

99.	Williams R (2011) pKa Data compiled by R.Williams. In: Res. psu.edu/brpgroup/pKa_compilation.pdf. http://research.chem.psu.edu/brpgroup/pKa_compilation.pdf. Accessed 28 Oct 2022

100.	Aggarwal VK, Emme I, Fulford SY (2003) Correlation between pKa and reactivity of quinuclidine-based catalysts in the Baylis-Hillman reaction: Discovery of quinuclidine as optimum catalyst leading to substantial enhancement of scope. J Org Chem 68:692–700. https://doi.org/10.1021/jo026671s
